# Protocol for culturing healthy primary endothelial cells isolated from research-grade human corneoscleral donor tissue

**DOI:** 10.1016/j.xpro.2026.104535

**Published:** 2026-04-30

**Authors:** Jeffrey M.A. van der Krogt, Marchien G. Dallinga, Antoon J. van den Bogaerdt, Ivanka J.E. van der Meulen, Jaap D. van Buul

**Affiliations:** 1 Department of Medical Biochemistry, Amsterdam UMC, 1105 AZ Amsterdam, the Netherlands; 2 Department of Ophthalmology, Amsterdam UMC, 1105 AZ Amsterdam, the Netherlands; 3 Department of Ophthalmology, UMC Utrecht, 3584 CX Utrecht, the Netherlands; 4 Cornea Department, ETB-BISLIFE, 2031 CG Haarlem, the Netherlands; 5 Leeuwenhoek Centre for Advanced Microscopy at Swammerdam Institute for Life Sciences, University of Amsterdam, 1098 XH Amsterdam, the Netherlands

**Keywords:** Cell culture, Cell isolation, Microscopy

## Abstract

Studying the function of healthy corneal endothelial cells has been challenging due to a variety of culturing strategies. Here, we present a standardized protocol for culturing primary human corneal endothelial cells, isolated from research-grade corneoscleral donor tissue, without the addition of mitotic enhancers. We describe the required enzymes, medium composition, cell density, and incubation times. Additionally, we use immunofluorescence microscopy to illustrate optimal outcomes and discuss troubleshooting procedures.

## Highlights

• Steps for isolating and culturing endothelial cells from corneoscleral donor tissue
• Instructions to prevent corneal endothelial-to-mesenchymal transition in culture
• Guidance on the assessment of corneal endothelial cell health in culture

## Abstract (editor highlights)

Publisher’s note: Undertaking any experimental protocol requires adherence to local institutional guidelines for laboratory safety and ethics.

## Graphical abstract

**Figure:**
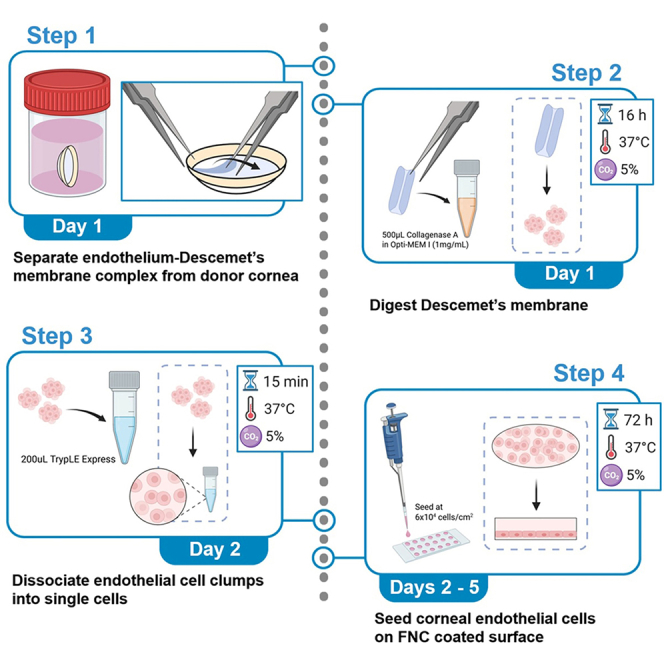

## Before you begin

The corneal endothelium functions as a selective barrier to regulate the movement of solutes from the anterior eye chamber into the corneal stroma. By means of the so-called “pump-and-leak” mechanism, the endothelium tightly regulates this diffusion of solutes while at the same time actively pumping water in the opposite direction to keep the cornea in a slightly dehydrated state that is required for optical transparency.^1^

Corneal endothelial cells (CECs) have no mitotic activity. The CEC density decreases from approximately 3.5 × 10^5^ cells/cm^2^ at birth to an average cell density of 3.1 × 10^5^ cells/cm^2^ at age 20 and continues to decrease with a rate of 0.4%–0.6% annually in healthy individuals.^2^ This decrease in cell density with age is accelerated by trauma (including surgery), inflammation, and degenerative diseases of the cornea such as Fuchs’ endothelial dystrophy. After a mild decrease in cell density, remaining CECs expand their surface (polymegathism) and change shape (pleiomorphism) to secure the endothelial barrier function. However, when the cell density falls below 5.0 × 10^4^ cells/cm^2^, the remaining cells can no longer compensate for the loss, resulting in corneal edema and loss of vision. In ophthalmology clinics, patients with corneal edema due to low CEC density are regularly seen.

### Innovation

In order to better understand the mechanisms through which healthy endothelial cells rescue their barrier function prior to corneal decompensation, a standardized protocol for culturing a healthy CEC monolayer is needed. Over the past decade, numerous protocols for culturing CECs have been developed for innovative cell therapy purposes. These protocols include the use of mitotic enhancers such as growth factors (e.g., epidermal growth factor^3^) and cell signaling reagents (e.g., ROCK inhibitor Y-27632^4^). Despite promising results for cell therapy, stimulating cell proliferation in CECs faces the challenge of inducing endothelial-to-mesenchymal transition and an excess of mitotic enhancers has been found to negatively impact cell survival, proliferation, and morphology.^5^ Therefore, in the current protocol, we provide the specific steps needed for culturing a monolayer of functional primary human CECs isolated from corneoscleral donor tissue, without the use of mitotic enhancers. We make use of human corneoscleral donor tissue that has been rejected for transplantation due to a low endothelial cell count or a high variety in endothelial cell size but still contains healthy CECs. Throughout this article we refer to this tissue as research-grade corneoscleral donor tissue.

In addition to omitting mitotic enhancers, additional improvements of the current protocol over existing CEC culturing protocols include (1) optimization of the cell seeding density, (2) application of a low centrifugation speed, (3) no restrictions on donor age, sex or ethnicity and (4) enabling longer preservation times of the corneoscleral donor tissue up to a maximum of 30 days.

### Institutional permissions

This study was performed in compliance with the Declaration of Helsinki. Human research-grade corneoscleral donor tissue, from Dutch post-mortem donors who had consented to research, was provided by ETB-BISLIFE: Multi Tissue Center, Haarlem, The Netherlands, conform Dutch national donor regulations. Experiments were performed in accordance with Dutch national regulations. All applied protocols were approved by the Amsterdam UMC ethics review committee for non-WMO research (METC 2024.0496). If applicable, scientists using the current protocol should still acquire permissions from their local institutions.

### Storage of the corneoscleral donor tissue

**Timing: 10 min**

1. Submerge the corneoscleral donor tissue in preservation medium.
  ***Note:*** The inclusion of research-grade corneoscleral donor tissue does not impose any restrictions to donor age, sex, or ethnicity.
  a. Add 40 mL preservation medium to a 60 mL sterile sample container.
  b. Position the corneoscleral donor tissue vertically inside the preservation medium to avoid accumulation of debris on the endothelium.
    ***Note:*** This can be achieved either by attaching a floating device to the peripheral scleral rim of the corneoscleral donor tissue or by placing a 10-0 ethilon suture through the scleral rim and attaching it to the lid of the sample container.^6^
2. Store the corneoscleral donor tissue.
  a. Firmly apply Parafilm M laboratory film on top of the sample container and leave the screw cap off to allow for gas exchange without moisture loss.
  b. Place the cornea in the incubator and store at 37°C and 5% CO_2_ for a maximum of 30 days.

***Note:*** Parafilm M laboratory film is permeable to gases but waterproof, making it ideal for protecting the contents of a sample container without causing a hypoxic environment.

***Note:*** The Parafilm M laboratory film is not sterile and contact with the preservation medium should therefore be avoided to minimize the risk of infection. The addition of penicillin and streptomycin to the applied media further lowers this risk.

## Key resources table

**Table:** 

REAGENT or RESOURCE	SOURCE	IDENTIFIER
**Antibodies**		

Anti-human ZO-1 antibody	BD Biosciences	Cat. No.: 610966
Anti-human ATP1A1 antibody	Proteintech	Cat. No.: 55187-1-AP
Anti-human N-cadherin antibody	Cell signaling technology	Cat. No.: 13116
Anti-human CD56 (NCAM) antibody	R&D systems	Cat. No.: MAB24083
Anti-human CD166 antibody	BioLegend	Cat. No.: 343902
Anti-human Ki67 antibody	Abcam	Cat. No.: ab16667
Chicken anti-Mouse IgG (H+L) AF488	Invitrogen	Cat. No.: A-21200
Donkey anti-Rabbit IgG (H+L) AF568	Invitrogen	Cat. No.: A10042
Chicken anti-Rabbit IgG (H+L) AF594	Invitrogen	Cat. No.: A21442

**Biological samples**		

Research-grade human corneoscleral donor tissue	ETB-BISLIFE, Haarlem, The Netherlands	https://www.etb-bislife.org/

**Chemicals, peptides, and recombinant proteins**		

Collagenase A from Clostridium histolyticum	Roche	Cat. No.: 10103578001
FNC Coating Mix	Athena Enzyme Systems	Cat. No.: 0407
TrypLE Express	Gibco	Cat. No.: 12604013
Minimal Essential Medium	Gibco	Cat. No.: 11095080
Opti-MEM I with GlutaMAX supplement	Gibco	Cat. No.: 51985034
4-(2-hydroxyethyl)-1-piperazineethanesulfonic acid (HEPES)	Sigma-Aldrich	Cat. No.: H3375
Sodium bicarbonate	Sigma-Aldrich	Cat. No.: S6014
D-(+)-Glucose	Sigma-Aldrich	Cat. No.: G8270
L-glutamine	Sigma-Aldrich	Cat. No.: G7513
Sodium pyruvate	Gibco	Cat. No.: 11360070
Newborn calf serum	Gibco	Cat. No.: 26010074
Fetal bovine serum	Sigma-Aldrich	Cat. No.: F9665
Penicillin-Streptomycin	Sigma-Aldrich	Cat. No.: P0781
Amphotericin B	Gibco	Cat. No.: 15290026
4% Paraformaldehyde solution in PBS	TebuBio	Cat. No.: AR1068
Fetal bovine Serum Albumin	Sigma-Aldrich	Cat. No.: F9665
Dulbecco’s Phosphate-buffered Saline	Gibco	Cat. No.: 14190169
Mounting medium	Ibidi	Cat. No.: 50001
Endothelial cell growth medium 2	PromoCell	Cat. No.: C-22211
EGM2 Supplement Mix	PromoCell	Cat. No.: C-39216
TC20 Automated Cell Counter	Bio-Rad	Cat. No.: 1450102

**Software and algorithms**		

ImageJ/FIJI	Schneider C.A. et al.^7^	https://imagej.net/ij/
ZEN Imaging Software	ZEISS	N/A
LAS X Life Science Microscope Software	Leica Microsystems	N/A

**Other**		

Straight toothed forceps	Malosa	Cat. No.: 1124
Curved tying forceps	Hasa Optix	Cat. No.: 551175
Sinskey hook	Malosa	Cat. No.: 654121
DMEK guarded punch	Moria	Cat. No.: 17207Dxxx
Screw cap micro tube, 2 mL	Sarstedt	Cat. No.: 72.694
Sterile straight container, 60 mL	Corning Gosselin	Cat. No: TP35C-002
Parafilm M laboratory film	Amcor	Cat. No.: 11772644
DMi8 automated inverted epifluorescence microscope	Leica Microsystems	Item No.: 11889113
DFC9000 GT sCMOS camera	Leica Microsystems	Ser. No.: VSC-09334
M80 routine stereo microscope	Leica Microsystems	Item No.: 10450155
Primovert inverted microscope	ZEISS	Item No.: 415510-1105-000
Dual-Chamber counting slides	Bio-Rad	Cat. No.: 1450011
30 mm × 0.45 μm cellulose acetate syringe filter	Cytiva Whatman	Cat. No.: 10462100
10-0 Nylon suture	Ethilon	Cat. No.: W1768
Corneosceral donor tissue floating device	Lie J.T. et al.^6^	https://doi.org/10.1136/bjo.2008.140574

## Materials and equipment

**Table:** Preservation medium

Reagent	Final concentration	Amount
Minimal Essential Medium	N/A	98 mL
4-(2-hydroxyethyl)-1-piperazineethanesulfonic acid	25 mmol/L	595.753 mg
Sodium bicarbonate	26 mmol/L	218.418 mg
D-(+)-Glucose	5.5 mmol/L	99.086 mg
L-glutamine	2 mmol/L	29.228 mg
Pyruvate	1 mmol/L	8.806 mg
Newborn calf serum	2%	2 mL
Penicillin	10 IU/mL	1,000 IU
Streptomycin	0.1 mg/mL	10 mg
Amphotericin B	0.25 μg/mL	25 μg
**Total**	**N/A**	**100 mL**

Store at −20°C for up to 3 months.

**Table:** CEC culture medium

Reagent	Final concentration	Amount
Opti-MEM I with GlutaMAX supplement	N/A	95 mL
Fetal bovine serum	5%	5 mL
Penicillin	100 IU/mL	10,000 IU
Streptomycin	100 μg/mL	10 mg
Amphotericin B	0.25 μg/mL	25 μg
**Total**	**N/A**	**100 mL**

Store at 4°C for up to 3 months.

**Table:** Endothelial cell growth medium 2 (EGM2)

Reagent	Final concentration	Amount
EGM2 basal medium	N/A	487 mL
EGM2 Supplement Mix	N/A	13 mL
Penicillin	100 IU/mL	10,000 IU
Streptomycin	100 μg/mL	10 mg
Amphotericin B	0.25 μg/mL	25 μg
**Total**	**N/A**	**500 mL**

Store at 4°C for up to 3 months.

**Table:** Collagenase A, 1 mg/mL

Reagent	Final concentration	Amount
Opti-MEM I with GlutaMAX supplement	N/A	500 μL
Collagenase A from Clostridium histolyticum	1 mg/mL	0.5 mg
**Total**	**N/A**	**500 μL**

Store at −20°C for up to 1 week.

## Step-by-step method details

### Separate the endothelium-Descemet’s membrane complex from the corneoscleral donor tissue

**Timing: 45 min preparation, 16 h incubation**

This step describes the procedure in which the endothelium-Descemet’s membrane (EDM) complex is separated from the corneoscleral tissue (descemetorhexis). This enables the isolation of CECs without contamination from other cell types (e.g., corneal stromal or epithelial cells).

1. Prepare Collagenase A, 1 mg/mL.
  a. Prepare 500 μL Collagenase A, 1 mg/mL according to the recipe displayed in the “materials and equipment” section.
  b. In a flow cabinet, filter sterilize the dissolved Collagenase A using a 0.45 μM pore size membrane filter.
  c. Add 500 μL sterilized Collagenase A to a screw cap micro tube and keep the tube at room temperature (RT).
2. Collect sterile surgical tools needed for the descemetorhexis (straight toothed forceps; curved tying forceps; Sinskey hook and DMEK guarded punch cup).
3. Take the corneoscleral donor tissue from the incubator and remove the Parafilm M laboratory film from the sample container.
4. Install the surgical setup.
  a. Set the routine stereo microscope to a 1.25× magnification and turn on the microscope light.
  b. Place the DMEK guarded punch cup at a central position of the microscope.
  c. With straight toothed forceps, grab hold of the floating device or the suture and lift the corneoscleral donor tissue out of the sample container.
  d. Remove the floating device or the suture from the corneoscleral donor tissue.
  e. Place the corneoscleral donor tissue endothelial side facing upwards in the center of the DMEK guarded punch cup.

***Note:*** Do not entirely remove preservation medium from the corneoscleral donor tissue as this will negatively affect the tissue surface tension.

5. Separate the EDM complex from the corneoscleral donor tissue (Methods video S1).
  a. Hold the straight toothed forceps in your non-operating hand and use it to stabilize the corneoscleral donor tissue.
  b. Hold the Sinskey hook in your operating hand and place the tip of the hook directly central to the Schwalbe line.
  c. In a fluent motion and with retained force, make a circumferential incision to isolate the EDM complex from the peripheral trabecular meshwork.
    ***Note:*** Frequently adjust the position of the straight toothed forceps to keep the corneoscleral donor tissue in optimal position for descemetorhexis.
    ***Note:*** The goal here is to separate the EDM complex from the trabecular meshwork to enable stripping of the EDM complex from the corneoscleral donor tissue. Applying too much force on the incision causes the Sinskey hook to be stuck in the corneal stroma, while applying too little force leaves the Descemets’ membrane intact.
  d. Using the curved tying forceps, gently grab a peripheral edge of the isolated EDM complex and carefully strip the EDM complex towards the opposite direction (Figure 1).
    ***Note:*** It is not necessary to separate the EDM complex from the corneoscleral donor tissue in one piece (troubleshooting 1).

**Figure 1:**
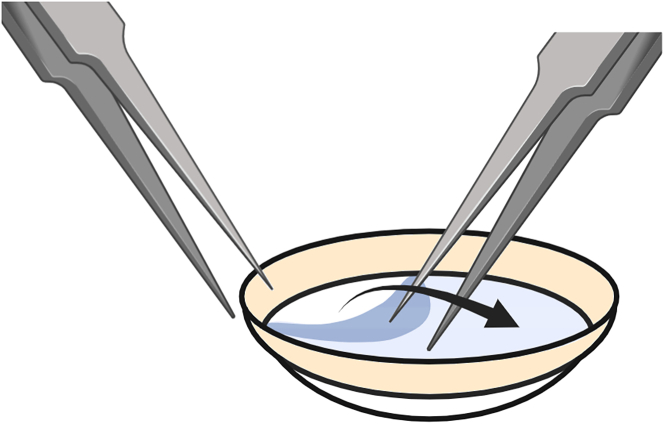
Descemetorhexis procedure A schematic illustration of the procedure in which the EDM complex is separated from the peripheral corneoscleral structures. Created in Biorender.com.
6. Dissolve the Descemet’s membrane.
  a. Transfer the separated EDM complex into the screw cap micro tube and submerge it in 500 μL Collagenase A, 1 mg/mL.
  b. Firmly apply Parafilm M laboratory film on top of the screw cap micro tube and leave the screw cap off. Incubate the endothelium at 37°C and 5% CO_2_ for sixteen hours.

### Dissociate the corneal endothelium into single corneal endothelial cells

**Timing: 40 min**

This step describes the procedure in which clumps of CECs that have been formed after digesting the Descemet’s membrane are dissociated into single cells ready to be seeded.

7. Dissociate the cell clumps into a single cell suspension.
  a. Take the screw cap micro tube from the incubator and resuspend the endothelium using a 1000 μL pipette tip to dissociate the endothelium into large cell clumps (troubleshooting 2).
  b. Centrifuge the micro tube at 200 × *g* for 6 min at RT to pellet the CECs.
  c. Carefully remove the supernatant, add 200 μL pre-warmed TrypLE Express onto the cell pellet (troubleshooting 3).
    ***Note:*** Make sure the cell pellet comes loose from the inner side of the tube when adding TrypLE Express, resuspending is not needed here.
  d. Incubate the cell suspension at 37°C and 5% CO_2_ for 15 min in total. After 7.5 min, tap against the micro tube to loosen the cell clumps.
8. Inactivate the TrypLE Express and acquire the preferred cell density.
  a. Add 300 μL pre-warmed CEC culture medium and resuspend to dissociate the cell aggregates into single cells.
  b. Centrifuge the micro tube at 200 × *g* for 6 min at RT to pellet the CECs.
  c. Carefully remove the supernatant and resuspend the pellet in 250 μL pre-warmed CEC culture medium to obtain a single cell suspension.
  d. Count the CECs in suspension (applying a cell size range between 6–30 μm).
    ***Note:*** Cell counting was performed using the Bio-Rad TC20 Automated Cell Counter with Bio-Rad Dual-Chamber counting slides (Bio-Rad, Hercules, CA, USA).
  e. Centrifuge the micro tube at 200 × *g* for 6 min at RT to pellet the CECs.
  f. In the meantime, calculate the volume of CEC culture medium required to obtain a cell seeding density of 6 × 10^4^ cells/cm^2^.
    ***Note:*** Imagine the total CEC yield obtained from one corneoscleral donor tissue equals 8 × 10^4^ cells cells. The intended cell culture surface area equals 0.32 cm^2^ (96 well plate) and the preferred seeding volume for this culture surface equals 100 μL. In order to get the preferred cell seeding density of 6 × 10^4^ cells/cm^2^ (1.92 × 10^4^ cells in one well of 0.32 cm^2^) onto the culture surface, resuspend the CEC pellet in 416.67 μL ((8 × 10^4^ / 1.92 × 10^4^) × 100 μL).
    ***Note:*** On average 8 × 10^4^ CECs (range 4 × 10^4^ – 12 × 10^4^) are isolated from one corneosceral donor tissue. The minimal cell density that is needed for optimal CEC culture is 6 × 10^4^ cells/cm^2^, meaning that a cornea with an average endothelial cell yield is to be cultured on a surface area of maximal 1.33 cm^2^.
  g. Carefully remove the supernatant and resuspend the pellet in pre-warmed CEC culture medium of the calculated volume to obtain a single cell suspension.

### Seed the corneal endothelial cells onto an FNC-coated surface

**Timing: 20 min**

This step describes the procedure in which CECs are seeded, as well as the procedure to create and preserve a healthy monolayer.

9. Coat the cell culture surface with FNC Coating Mix.
  a. Place the sterile plastic cultureware in a flow cabinet.
  b. Add FNC Coating Mix onto the cell culture surface. Depending on the type of cultureware used, the amount of FNC Coating Mix will vary between 80 – 250 μL/cm^2^. The surface should be entirely covered.
  c. Incubate for 30 s at RT and remove the FNC Coating Mix with a pipet.

***Note:*** For a detailed protocol on FNC Coating Mix, see https://athenaes.com/PromotionalMaterials/Promotional%20Materials/FNCFlier%20wdatasheet%20v2017.pdf.

10. Seed the corneal endothelial cells.
  a. Gently resuspend the CEC suspension, aspirate the preferred seeding volume for the intended culture surface and place the pipette tip on a bottom edge of the cell culture surface.
  b. Add the cell suspension onto the coated surface and gently resuspend three times to equally distribute the CECs (troubleshooting 4).
  c. Confirm homogeneous cell distribution using an inverted microscope with 4x magnification and incubate the cells at 37°C and 5% CO_2_ for 90 min.
11. Carefully refresh the CEC culture medium 90 min after seeding, and once every 12–48 h thereafter, depending on the well volume.

***Note:*** Minimize the exposure of the CECs to shear stress, since this may induce endothelial-mesenchymal transition.

***Note:*** Always check CEC confluency before and after refreshing the medium and, after 72 h of culture, confirm the formation of an intact monolayer with hexagonal shaped cells using an inverted microscope with 4× magnification (troubleshooting 5).

### General procedures

#### Microscopy

Phase-contrast images were obtained using a Axio Observer microscope (ZEISS, Oberkochen, Germany) with a 10× air objective (ZEISS, 420341-9911, Plan-Neofluor 10× / 0.3 Ph1). The microscope was operated using the ZEN Imaging Software (ZEISS, Oberkochen, Germany).

Immunofluorescence (IF) images were acquired using an inverted Leica DMI8 widefield microscope with a motorized XY stage ITK QUANTUM, speed 500 mm/sec, accuracy < ±1 μm. Used objectives include the HC PL Fluotar 20× / 0.40 dry objective for Ki67, the HC PL Fluotar 40× / 0.60 dry objective for ZO-1 and the HC PL APO 63× / 1.40 oil objective for ATP1A1, N-cadherin, CD56 and CD166. CECs labeled with Alexa Fluor 488 (Invitrogen Cat. #: A-21200) were excited using LED 475 with a BP510/40 emission filter (power 60%; zero gain; exposure time 300 ms). CECs labeled with Alexa Fluor 568 (Invitrogen Cat. #: A10042) were excited using LED 555 with a BP590/50 emission filter (power 60%; zero gain; exposure time 300 ms). CECs labeled with Alexa Fluor 594 (Invitrogen Cat. #: A-21442) were excited using LED 575 with a 100% emission filter (power 60%; zero gain; exposure time 300 ms). Fluorescence signal was detected using the Leica DFC9000 GT sCMOS camera with a front-illuminated scientific CMOS CIS2020A sensor, 50 fps acquisition speed, chip size 2048 × 2048, pixel size 6.5 mm, 16 bit. The microscope was operated using the Leica LAS X Life Science Microscope Software (Leica Microsystems, Wetzlar, Germany). An overview of the applied primary and secondary antibodies is provided in the key resources table.

**Figure 2:**
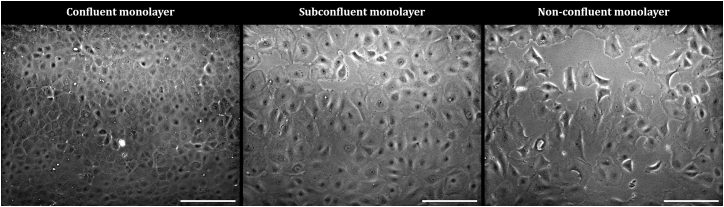
Seeding density affects CEC confluence Phase-contrast images of CECs 72 h after seeding on a FNC coated plastic surface with different cell densities (left: 6.5x10^4^ cells/cm^2^; middle 4.5x10^4^ cells/cm^2^; right: 2.5x10^4^ cells/cm^2^). Scale bar: 200 μm.

**Figure 3:**
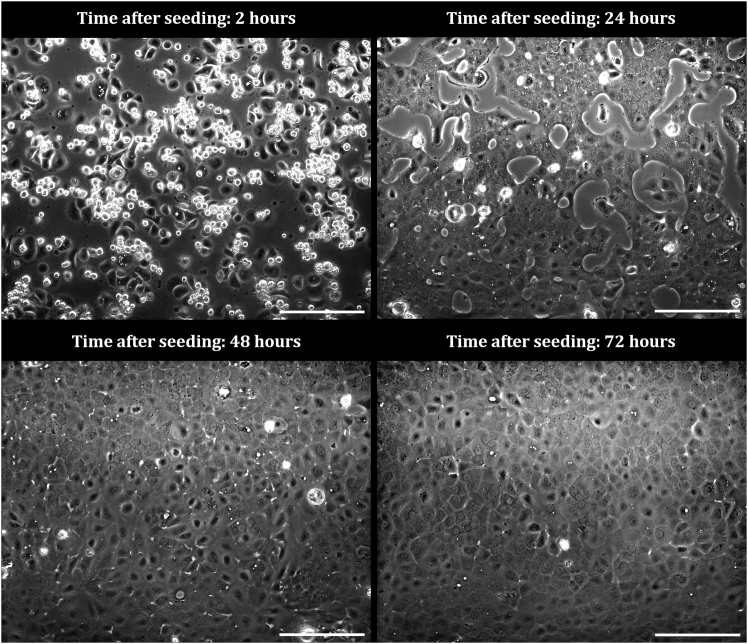
CECs form an intact monolayer 72 h after seeding Phase-contrast images of CECs seeded on a FNC coated plastic surface with a cell density of 6.5x10^4^ cells/cm^2^; 2 h, 24 h, 48 h and 72 h after seeding. White, round structures represent adhered CECs that have not yet flattened out across the bottom surface. Scale bar: 200 μm.

All obtained images were processed and analysed with the use of ImageJ/FIJI. Light-path disturbances of phase-contrast images shown in Figures 2 and 3 were corrected using the rolling ball background correction plugin in FIJI. This overview of technical properties for IF imaging was generated following the best practices guideline provided by Montero Llopis and co-workers (2021).^8^

#### Cell treatment

To determine the minimal cell seeding density and duration necessary for a functional CEC monolayer, CECs were seeded in a μ-Slide 15 well 3D chambered coverslip (Ibidi, Gräfelfing, Germany) at decreasing density (6.5 × 10^4^ cells/cm^2^; 4.5 × 10^4^ cells/cm^2^; and 2.5 × 10^4^ cells/cm^2^ for a duration of 72 h (Figure 2). Phase-contrast images were obtained at 2 h, 24 h, 48 h and 72 h after seeding (Figure 3). Corneoscleral donor tissue applied during this experiment were derived from a 83-year-old male donor and were preserved for 25 days in preservation medium before use.

To quantify the spatial expression of functional proteins, CECs were seeded in a μ-Slide 15 well 3D chambered coverslip (Ibidi, Gräfelfing, Germany) at a cell density of 6.5 × 10^4^ cells/cm^2^ for 72 h, after which the cells were prepared for IF imaging. CECs were fixed with 4% paraformaldehyde (PFA) solution in phosphate-buffered saline (PBS) for 12 min at 37′C, followed by washing in PBS and blocking in 2% bovine serum albumin (BSA) in PBS for 45 min at 37′C. CECs were incubated with primary antibodies dissolved in 0.5% BSA in PBS for 60 min at 37′C. After washing in PBS, CECs were incubated with secondary antibodies for 45 min at 37′C. Cells were mounted using mounting medium (Ibidi, Gräfelfing, Germany). Corneoscleral donor tissue applied during this experiment were derived from a 88-year old female donor and were preserved for 28 days in preservation medium before use.

Mitotic activity of cultured CECs was determined through the expression of Ki67, a protein strictly associated with cell proliferation. Three groups of cells were grown for 24 h, including (1) human umbilical vein endothelial cells (HUVECs) as a positive control for cell proliferation, (2) CECs cultured without the addition of mitotic enhancers and (3) CECs cultured with 10 μM Y-27632 (Figure S1). HUVECs were seeded at 3.0 × 10^4^ cells/cm^2^ and cultured in EGM2. CECs were seeded at 6.0 × 10^4^ cells/cm^2^ and cultured in CEC culture medium either with or without 10 μM Y-27632 respectively for 24 h. At 24 h after seeding and reaching 60%–80% confluency, cells were fixed with 4% PFA solution in PBS at 37′C (six minutes for HUVECs and twelve minutes for CECs), followed by washing in PBS and permeabilization with 0.1% Triton-X100. Cells were then washed in PBS and blocked in 2% bovine serum albumin (BSA) in PBS for 45 min at 37′C. Primary antibodies dissolved in 0.5% BSA in PBS were added for 60 min at 37′C. After washing in PBS, cells were incubated with secondary antibodies for 45 min at 37′C and mounted using mounting medium (Ibidi, Gräfelfing, Germany). In line with the findings of Bartakova and colleagues, the addition of 10 μM Y-27632 to the CEC culture medium did not significantly increase mitotic activity.^5^ Corneoscleral donor tissue applied during this experiment were derived from an 86-year-old female donor and were preserved for 15 days in preservation medium before use.

## Expected outcomes

**Figure 4:**
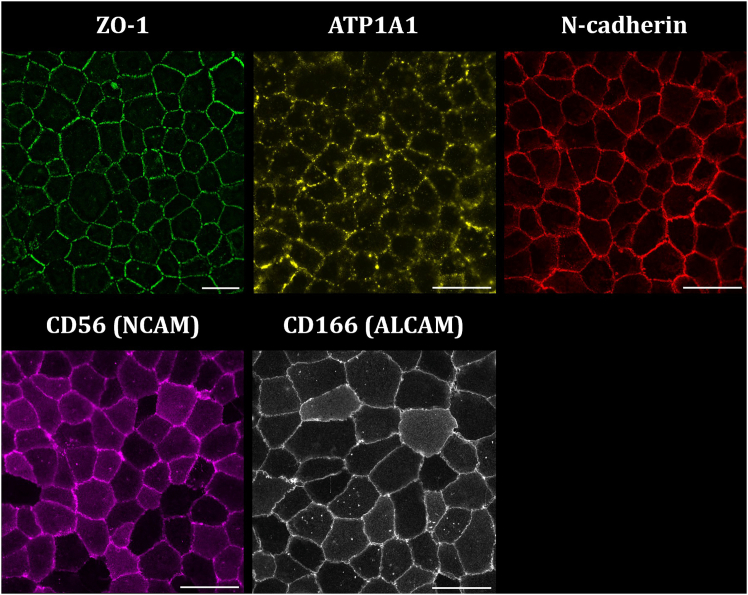
A panel of five proteins characteristic for the healthy CEC IF images of CECs 72 h after seeding on a FNC coated plastic surface with a cell density of 6.5 × 10^4^. Scale bar: 50 μm.

The primary objective of this protocol is to create a monolayer of functional primary CECs, isolated from research-grade human corneoscleral donor tissue without the use of mitotic enhancers. Seeding the CECs in a minimal density of 6 × 10^4^ cells/cm^2^ for a minimal duration of 72 h according to the guidelines of the current protocol generates a CEC monolayer with complete characteristics of healthy, functional CECs. These characteristics include (1) a hexagonal ‘cobblestone-like’ morphology (Figures 2 and 3), (2) a panel of five proteins characteristic for CEC function, including ZO-1, ATP1A1, N-cadherin, CD56 (NCAM) and CD166 (ALCAM)^9^ (Figure 4) and (3) a low mitotic activity (Figure S1).

## Limitations

This protocol has several limitations. First, due to the lack of mitotic activity in healthy CECs, the surface area that can be seeded with cells from one cornea with an average endothelial cell density is limited to 1.33 cm^2^. In addition, the number of CECs isolated from one research-grade corneoscleral donor tissue are to some extent reliant on the operator’s skills. However, following the current protocol, the procedure comes with a steep learning curve and can be adopted even by researchers with minimal to no surgical skills.

## Troubleshooting

### Problem 1

The Descemet’s membrane ruptures during separation of the EDM complex from the corneoscleral donor tissue (note after step 7 d).

### Potential solution

Place the ruptured part of the Descemet’s membrane on a central part of the corneoscleral donor tissue. With the Sinskey hook, confirm circumferential isolation of the EDM complex from the scleral rim. Using the curved tying forceps, gently grab hold of another peripheral edge of the EDM complex and continue to separate the EDM complex from the corneoscleral donor tissue.

### Problem 2

The EDM complex does not dissociate into smaller cell clumps after sixteen hours of incubation with Collagenase A, 1 mg/mL (step 9a).

### Potential solution

While resuspending, make sure the tissue clumps move through the small opening of the 1000 μL pipette tip to stimulate its dissociation into smaller cell clumps.

### Problem 3

The cell pellet comes loose during removal of the supernatant after centrifugation (step 9c).

### Potential solution

Return the aspirated supernatant into the screw cap micro tube and resuspend the pellet until no cell clumps are visible. Repeat the centrifugation step (200 × *g* for 6 min at RT) and carefully remove the supernatant.

### Problem 4

After seeding, the CECs are not homogeneously spread and large parts of the intended culture surface area are not covered by cells (step 12 b).

### Potential solution

Place an empty pipette tip on the bottom edge of the coated surface area and gently resuspend three times to create a homogeneous distribution of the CECs.

**CRITICAL:** Homogenize the CEC solution no more than 2 min after seeding since most of the CECs will have adhered to the FNC coated culture surface by then and redistribution becomes difficult.

### Problem 5

After 72 h, the CECs have not formed a confluent monolayer (second note after step 13; Figure 2 middle and right panel).

### Potential solution

It is likely that the CECs have been seeded in cell density below 6 × 10^4^ cells/cm^2^. Wash the surface area twice with 37°C PBS and cover the surface area with TrypLE Express (apply same amount used to coat the surface area with FNC Coating Mix). Incubate the cells for twelve to 25 min at 37°C and 5% CO_2_ and resuspend to dissociate the cells from their surface into a single CEC solution. From this point, add CEC culture medium to the CEC solution (1.5 times the volume of TrypLE Express) and repeat the culture protocol from Step 10 b onwards.

***Note:*** It is advised against using Trypsin-EDTA to dissociate the cells from their surface, since this may induce an irreversible fibroblastic appearance of the CEC.^10^

## Resource availability

### Lead contact

Further information and requests for resources and reagents should be directed to and will be fulfilled by the lead contact, Prof. Dr. Jaap D. van Buul (j.d.vanbuul@amsterdamumc.nl).

### Technical contact

Technical questions on executing this protocol should be directed to and will be answered by the technical contact, Dr. Jeffrey M.A. van der Krogt (j.m.vanderkrogt@amsterdamumc.nl).

### Materials availability

This study did not generate new unique reagents.

### Data and code availability

This study did not generate/analyze datasets or code.

## Acknowledgments

We would like to thank ETB-BISLIFE: Multi Tissue Center, Haarlem, the Netherlands for providing us with research-grade corneoscleral donor tissue. This research was in part funded by 10.13039/100016063UitZicht #2022-8. We also thank the Microscopy and Cytometry Core Facility of the Amsterdam UMC, location AMC, for providing access to their (advanced) microscopy facilities.

## Author contributions

Conceptualization, J.M.A.v.d.K., M.G.D., I.J.E.v.d.M., and J.D.v.B.; practical work, J.M.A.v.d.K. and M.G.D.; writing – original draft, J.M.A.v.d.K.; writing – review and editing, J.M.A.v.d.K., M.G.D., A.J.v.d.B., I.J.E.v.d.M., and J.D.v.B.; funding acquisition, M.G.D., A.J.v.d.B., I.J.E.v.d.M., and J.D.v.B.; supervision, M.G.D., A.J.v.d.B., I.J.E.v.d.M., and J.D.v.B. All authors agree to be accountable for all aspects of the work in ensuring that questions related to the accuracy or integrity of any part of the work are appropriately investigated and resolved.

## Declaration of interests

The authors declare no competing interests.

## Supplemental information

Document S1. Figure S1

Methods video S1. Descemetorhexis procedure, related to step 5 Video showing the surgical procedure in which the EDM complex is separated from the peripheral corneoscleral structures speed at 2x.
