# Supplementary material for: Protocol for culturing healthy primary endothelial cells isolated from research-grade human corneoscleral donor tissue
Source: STAR Protoc. 2026 Apr 30;7(2):104535. doi: 10.1016/j.xpro.2026.104535 (PMC13141704; doi:10.1016/j.xpro.2026.104535)
Supplement: Document S1. Figure S1 [file mmc1.pdf]

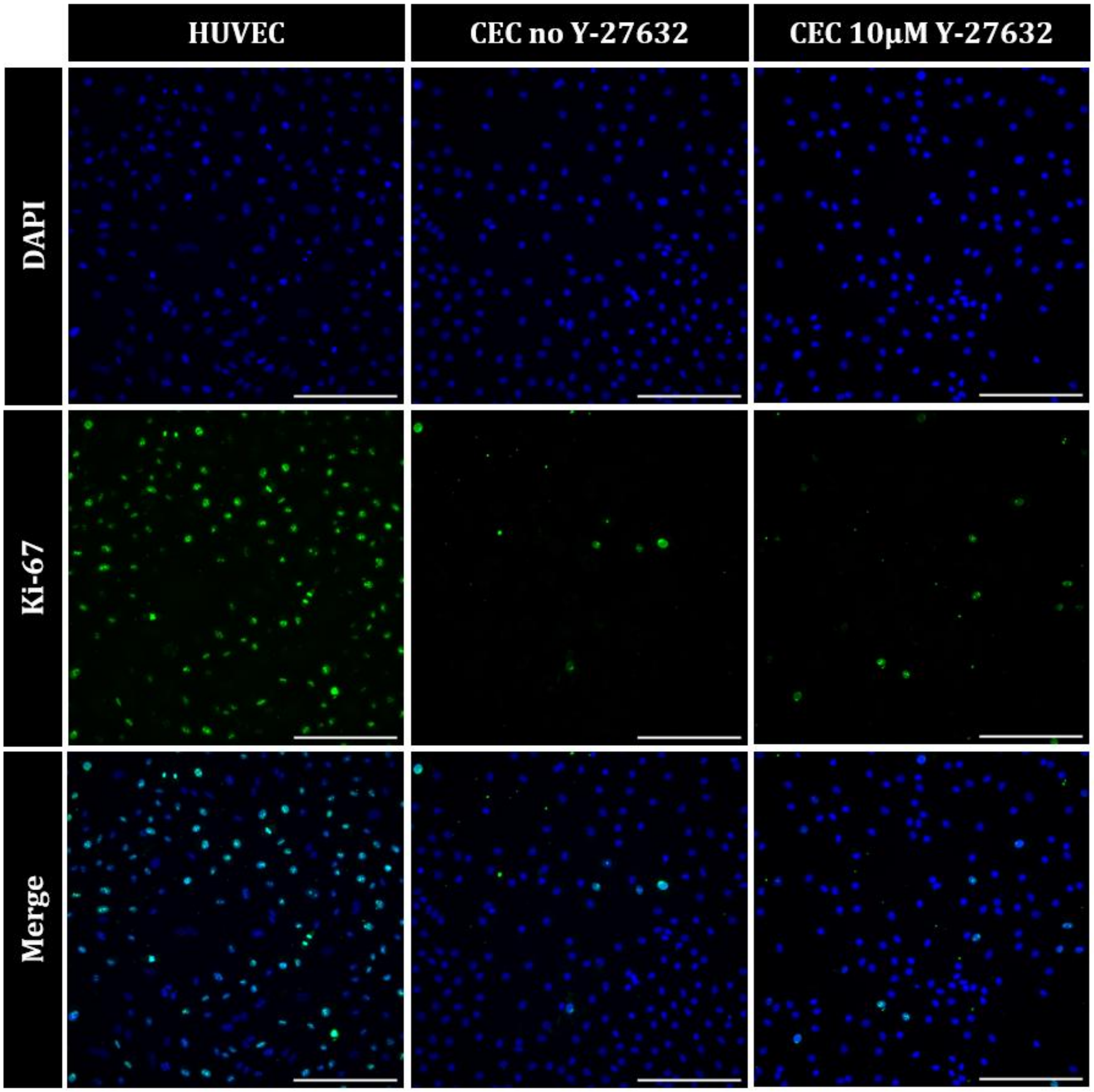

Supplemental Figure 1: **Limited mitotic activity in CECs.** IF images of HUVECs (left column), CECs without mitotic enhancers (middle column) and CECs with 10 µM Y-27632 (right column) showing limited mitotic activity in CECs cultured with or without a mitotic enhancer, as compared to HUVECs. Scale bar: 200 µm. Related to “Cell treatment” and “Expected outcomes” sections.
